# Multilayer Alginate Microcapsules For Live Cell Microencapsulation; Is There Any Preference For Selecting Cationic Polymers?

**DOI:** 10.22037/ijpr.2020.114096.14660

**Published:** 2021

**Authors:** Fariba Hajifathaliha, Arash Mahboubi, Noushin Bolourchian, Elham Mohit, Leila Nematollahi

**Affiliations:** a *Food Safety Research Center, Shahid Beheshti University of Medical Sciences, Tehran, Iran. *; b *Department of Pharmaceutics, School of Pharmacy, Shahid Beheshti University of Medical Sciences, Tehran, Iran. *; c *Department of Pharmaceutical Biotechnology, School of Pharmacy, Shahid Beheshti University of Medical Sciences, Tehran, Iran. *; d *Biotechnology Research Center, Pasteur Institute of Iran, Tehran, Iran.*

**Keywords:** Alginate, Cellulose sulfate, Poly l-lysine, Poly l-ornithine, Multilayer microcapsules, Cell microencapsulation

## Abstract

Since 1980 after introducing the concept of live cell encapsulation by Lim *et al.*, this technology has received enormous attention. Several studies have been conducted to improve this technique; different polymers, either natural or synthetic, have been used as microcapsules` making materials and different substances as coating layers. Literature review leads us to the conclusion that alginate (Alg) multilayer microcapsules and, in particular, alginate-poly l-lysine (PLL)-alginate (APA) are the most used structures for live cell encapsulation. Although, disadvantages of PLL (*e.g*., weak mechanical strength and low biocompatibility) made researchers work on other cationic polymers to find an alternative. This review aims to discuss more popularly suggested cationic polymers such as poly l-ornithine (PLO), chitosan, *etc*. As alternatives for PLL and, more importantly, we want to take a closer look to see which one of these systems are closer to clinical applications.

## Introduction

Microencapsulation technology in the pharmaceutical area has a very long history. In 1980, Lim *et al.* introduced a new application for this technique by encapsulating live cells into the alginate microcapsules. This technique includes immobilization of live cells in a polymeric semi-permeable membrane to reach different goals,*e.g*., protecting the encapsulated cells from the host immune system and keeping them functional for a prolonged time in the body (1). This concept became the motivation for many studies. They encapsulated pancreatic islets into the alginate microcapsules covered with PLL and poly ethylene imine (PEI); semi-permeable membrane which allows small molecules like glucose and nutrients to freely diffuse but prevents large molecules from passing through (2).

**Figure 1:**
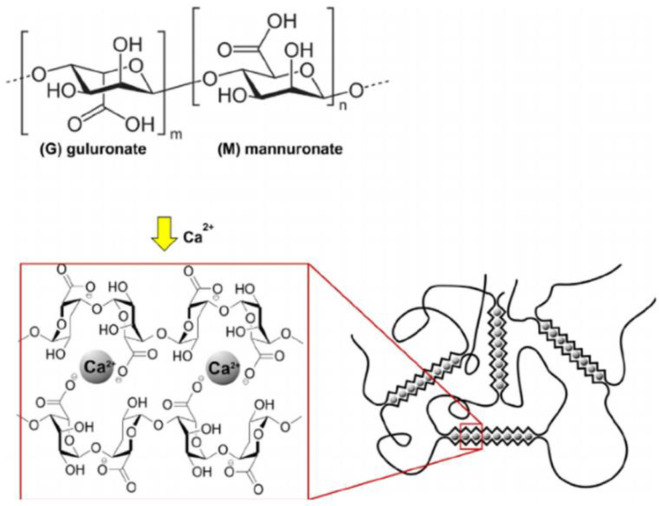
*Structure of alginic acid and egg-box model* ***(10)*** *.*

Different classes of polymers have been used as capsule-making materials. These include carbohydrates (agarose, carrageenan, chitosan, gellan gum, hyaluronic acid and alginate), proteins (gelatin, collagen, fibrin, elastin and silk protein) and synthetic polymers like poly hydroxyl ethyl methacrylate-methyl methacrylate, a copolymer of acrylonitrile and polyethylene glycol (3, 4). Since these different polymers have already been the subject of so many articles, we do not intend to discuss them. The literature review shows that alginate due to its high biocompatibility, easy and mild production process, and high gel-forming capacity is the most suitable polymer for cell microencapsulation (5). Interaction between cations, such as Ca^2+^, Sr^2+^, Ba^2+^, and carboxylate groups in alginic acid is known as the mechanism of alginate hydrogel production. These ionic interactions lead to the formation of a cross-linked hydrogel. As shown in Figure 1, this structure is called the egg-box model (6-9).

Several studies were carried out to improve alginate microcapsules. Some examples are comparing sodium alginate with different ratio of G/M and different molecular weight (11-13), different bivalent cationic cross-linkers (Ca^2+^, Ba^2+^, Sr^2+^) (13, 14), different pH and reaction times (15), liquid, semi-solid or solid core microcapsules (16, 17). 

Alginate hydrogel can absorb water and swells inliquids, which is the main reason for the instability of the alginate microcapsules. Therefore, to boost the structure of microcapsules, reduce their pore size and prevent them from being solubilized, they are covered with a cationic layer (*e.g*., PLL, PLO, chitosan, PEI, *etc*.) (18, 19). Despite these advantages, it has been shown that cationic surface charge could be immunogenic in biological systems. To overcome this issue, using another anionic layer (*e.g*., alginate) has been suggested to cover the positive surface charge, which leads to the formation of three-layer microcapsules (*e.g*., APA) (20, 21). 

PLL is the most used polycationic polymer in this area, and there are many studies in which APA has been used to encapsulate different kinds of cell lines (2, 22-25). Actually, due to the long history of research behind it, PLL has kind of become a standard polycation in the cell microencapsulation field. Literature review, however, shows that one of the main strategies applied to improve this technology is to replace PLL with another cationic polymer (natural or synthetic). Improving biocompatibility, mechanical strength, or cost-effectiveness of multilayer microcapsules are the reasons which are announced in different studies to replace PLL with other cationic polymers (19, 26). 

Some researchers were successful (27, 28) and some were not in finding alternative polymers for PLL (13, 16). Actually, in this area, we face a huge number of controversial studies. With the same cationic polymers being evaluated as a coating layer of microcapsules, opposite results have been reported. In what follows, we will briefly discuss some of these studies and their contradictive results. 

After so many years of study and publishing so many articles in this area, a good question might be why researchers are still working on PLL as a cationic layer for alginate microcapsules, despite the introduction of some better alternatives. Is it really necessary to try to exclude PLL from this technique? Or, more importantly, what is the role of these multilayer alginate microcapsules in biopharmaceutical industries? We will enter the issue by discussing some cationic polymers which are more popular as potential alternatives for PLL. Next, we will take a look at the live cell encapsulated systems which are closer to the clinical application. 

**Poly l-ornithine (PLO)**

PLO is a synthetic amino acid chain that is positively charged. Based on different studies, PLO is probably the most acceptable alternative for PLL. As a covering layer, it has been investigated in some small clinical trials (29), but these studies do not lead to the same conclusion. However, the other cationic polymers (e.g., chitosan) are not immune to such contradictions. 

A study conducted in 1996, the long-term stability of different types of alginate microcapsules coated with different polycationic polymers such as PLL, PDL or PLO was evaluated using the release of encapsulated blue dextran measurement. After a year of storage in saline solution, the minimum amount of blue dextran was released from the microcapsules coated with PLL, the authors related this to the highest stability (30). In contrast, when Darrabie *et al.* and Tam *et al.* compared PLO and PLL as covering layers for alginate microcapsules, they reported a smaller pore size and less swelling rate for the PLO-coated microcapsules. In complete agreement with other studies, the more efficient coverage with PLO than PLL has been attributed to its shorter amino acid structure (4, 19). The number of broken PLL-coated microcapsules at the end of the study (14 days) was significantly higher than PLO-coated microcapsules. Here, we need to mention that there are several studies in which APA microcapsules remained intact even after much longer periods of time(15, 19, 22). Anyhow, they concluded that PLO-coated microcapsules were mechanically more stable than PLL-coated microcapsules. Aside from better physicochemical properties, they also recorded less immunogenicity for PLO than PLL(14, 26).

In another research, Ponce *et al.* reached the opposite conclusion. In their study, they compared PLL, PDL and PLO as coating layer of alginate microcapsules. After *in-vitro* studies and also *in-vivo* evaluation of these different microcapsules in the peritoneal cavity of rats, they found that the minimum immune responses toward microcapsules were observed in PLL coated microcapsules. Therefore they suggested PLL as the best option to be used as microcapsules coating layer (13, 16). Thanos *et al.* investigated ultra-pure alginate microcapsules coated with PLO, implanted them in different sites of Long-Evans rat (*e.g*., brain, peritoneal cavity, and subcutaneous space). During 215 days, capsules were explanted and evaluated using Fourier-transform infrared spectroscopy (FTIR) and scanning electron microscopy (SEM). The results showed that the encapsulation technique produced stable microcapsules capable of survival in all sites for at least 215 days (27). In an *in-vivo* experiment, Porcine and human pancreatic islets were encapsulated in alginate microcapsules covered with PLL, then put in a vascular prosthesis and entered between the iliac artery and the contralateral vein of five diabetic dogs. The results showed that, without receiving any immunosuppressive therapy, the hyperglycemic situation was reversed in all dogs (in one dog completely and in the others partially) (31). The other study was conducted by the same group of scientists in 2006, it was actually a small clinical trial on 2 patients, and the efficacy of encapsulated human islets in alginate microcapsules coated with PLO and outer layer of alginate was investigated. They’ve had a pre-transplantation phase (72 h), in which, no morphological, functional or sterility changes were recorded. They reported, No side effects during or after the procedure. The authors concluded that even though they should continue their investigation on 8 more patients but the results of this study showed that this procedure is safe and without any specific side effect (29). In 2010, Khanna *et al.* encapsulated Fibroblast growth factor-1 (FGF-1) in alginate-PLO-alginate microcapsules and could record protein secretion up to 30 days successfully (32). The adapted microencapsulation procedure in this study, may be applicable for molecules like proteins but not for live cells (especially mammalian cells), because for coating of microcapsules, they suspended them for 30 min in PLO and for 45 min in alginate solutions. Mammalian cells cannot tolerate so long, out of their culture media and incubator. In a comprehensive study, Loh *et al.*, investigated the combinatorial effect of different parameters (*e.g*., G/M ratio in alginate, cross-linker type, different cationic polymer) on microcapsules properties. They concluded thatmicroencapsulation may determine which parameter affects microcapsules mostly and must be paid more attention. But they added based on their results, microcapsules coated with PLL, fabricated from 40/60 sodium alginate, cross-linked with barium chloride had the best mechanical and diffusion properties (12). 

**Chitosan**

Chitosan, a derivative of chitin (polysaccharide found in different insects and fungi), is a natural, biodegradable and non-toxic cationic polymer. The binding of chitosan to the alginate hydrogels is somehow irreversible. This binding is significantly stronger than alginate-PLL binding(33). The efficacy of chitosan as a coating layer for alginate microcapsules was investigated in several studies. In 2006, Baruch *et al.* evaluated four types of chitosan and pH and reaction time on microcapsules properties. They applied two methods of coating; (a) single-stage and (b) two-stage coating procedure. They showed that the single-stage coating procedure led to an increase of at least four times in cell viability than the two-stage procedure. Among different types of chitosan, applying high molecular weight (MW) chitosan glutamate and low (MW) chitosan chloride resulted in higher cell viability and suitable mechanical properties (15). In this study, the amount of cell viability which was recorded for two-stage coating method is surprisingly lower than single-stage method. This phenomenon could be due to the higher concentration of chitosan solution in this method in comparison with single-stage method (1% *vs.* 0.5%). Generally, the comparison would be more reliable if the conditions of the two coating methods (*e.g*., reaction time, concentration) were exactly the same. The reason is that, according to Zhang *et al.* who studied toxicity of different chitosan concentrations on mesenchymal stromal cell-loaded alginate-chitosan microcapsules, chitosan solutions with a concentration higher than 0.1% could be toxic and cause reduction in cell viability (34). The two methods of coating (single-stage vs two-stage) were investigated in another study, however, the results of this study were in favor of two-stage coating method, because the microcapsules which were coated with this method were mechanically stronger than single-stage coated microcapsules (35). Haque *et al.* have compared chitosan with PLL as two coating layers for encapsulation of liver cells. They claimed that alginate-chitosan (AC) microcapsules have several advantages compared to alginate-PLL (AP), but the only advantage they have shown for AC over AP was that the cell viability was higher in AC in comparison with AP after 30 days of storage at -80 °C, but if we take a closer look we will notice that the initial cell number in two different microcapsules was significantly different, and that makes the comparison difficult. However, they showed similar physical and appearance properties for AC and AP and higher cell viability in AP during 25 days in comparison with AC. In another effort, to boost the structure of alginate microcapsules, they have been covered with chitosan and then with genipin. Genipin is an extract of Gardenia fruits and recently has received more attention as an alternative cross-linker due to lower toxicity than chemical counterparts. During coating process, the microcapsules were suspended in chitosan solution for 30 min and after that for 24 h in genipin solution. As the authors have stated, spending this long time of coating procedure could be harmful for live cells and this procedure should be optimized in order to encapsulate them (36).

**Other cationic polymers**

Polyethyleneimine (PEI) is a cationic polymer that is compared frequently with PLL, mostly in gene delivery and has shown higher efficiency than PLL in that area (37). Previously we conducted a comprehensive comparison between PEI and PLL as coating layers for alginate microcapsules. That study aimed to introduce a cationic polymer which shows the physicomechanical properties like PLL and also is more cost-effective to be a preferable option for industrial scale-up purposes. According to our results, plain microcapsules coated either with linear PEI or with PLL showed similar *in-vitro* properties (19). Further studies were carried out to see the faith of CHO-K1 cells in Alginate-linear PEI-alginate (ALA) and APA microcapsules. Based on the results, linear PEI could be considered as a proper and more cost-effective alternative for PLL because not only did the ALA microcapsules show all the physicomechanical properties of the APA, but they also showed higher cell viability and metabolic activity (22). 

Wang *et al.* have introduced polyvinyl amine (PVA) as a better alternative for PLL in the fabrication of IW32 cells-loaded alginate microcapsules. The reason is that using PVA, microcapsules with better mechanical strength and permeability were fabricated. Also, the cell density and amount of secreted erythropoietin (EPO) were higher with PVA than PLL (28). In another study published by this research team, three cationic polymers (PLL, PVA and poly allylamine) were compared again as alginate microcapsules’ coating layers. In contrast to the previous study, they concluded that microcapsules coated with poly allylamine had similar strength to the APA, while microcapsules coated with PVA had weaker structure than APA (38). 

**Applications of cell microencapsulation technique**

Obviously, the purpose of all these efforts was to optimize the microencapsulated systems to play their roles in biopharmaceutical industries and clinics. We could categorize the application of these cell-loaded microcapsules into two main classes: *in-vitro* application and *in-vivo* application. 

***In-vitro*** **application**

*In-vitro* application of cell microencapsulation technology has also reached industrial-scale production. The first industrial application of cell microencapsulation (ENCAPSEL^®^) was the massive production of monoclonal antibodies. It was hybridoma cells encapsulated in alginate microcapsules coated with PLL. Using this technique, higher concentration and purity wereachieved in comparison with conventional cell culture methods (39, 40). The concept is simple, during encapsulation procedure, cells expressing monoclonal antibodies are encapsulated in microcapsules with biocompatible materials under mild conditions. Then they are transferred into the reactor containing cell culture medium to be provided with nutrients at a controlled temperature (41).

***In-vivo*** **application**

Using cell-loaded microcapsules to manage different disorders in the body has been an ambitious desire that recently got a lot closer to reality. 

Among all the different encapsulated systems, we can name someof them which have passed the preclinical studies and are currently undergoing clinical trials. Here, we will discuss them.

**Diabetes mellitus**

Undoubtedly, type 1 diabetes (T1D) is the main target of studies in the cell microencapsulation area. In T1D, beta islet cells of the pancreas (the insulin-producing cells) have been permanently destroyed by an autoimmune disease. Therefore, encapsulated insulin-producing cells could be a solution for this situation. 

Any microencapsulated system for usein diabetic patients, should pass very rigorous preclinical and clinical trials. These processes are truly time-consuming, and many projects would fail to reach the goals set by regulatory agencies. Fortunately, some microencapsulated systems passed the first steps of clinical trials and waited for the next levels.

Living Cell Technologies (LCT), in a joint venture with Otsuka Pharmaceutical Factory, are working on LCT’s DIABECELL^®^. DIABECELL^®^ is a xenotransplantation strategy based on the immunoisolation of islet cells derived from pathogen-free pigs within alginate microcapsules coated with PLO layer. Up to now, DIABECELL^®^ has been evaluated in phase I and II clinical trials on 46 patients in New Zealand, Argentina and Russia. Results showed that using DIABECELL^®^, hypoglycemic episodes that diabetic patients experienced and daily insulin dose were reduced (42-44). Contrary to critics arguing against the sufficiency of the clinical trials, Diabecell^®^ has been approved for sale in Russia in 2010 (45, 46). 

ViaCyte is another company that has invested in T1D. They want to conduct phase I and II clinical trials to evaluate the safety and efficacy of VC-01 (47). VC-01 is differentiated from human embryonic stem cells. These cells are supposed to be delivered to the body using Encaptra^®^ - a drug delivery device, which implants under the skin (48, 49). 

PharmaCyte Biotech, in partnership with Austrianova, are the other important investors in this field. They have developed Cell-in-a-Box technology. It is somehow different from other live cell microencapsulation technologies. Because, in this technology, the main material for the fabrication of microcapsules is cellulose sulphate, while the others have mostly used alginate. Melligen cells derived from the human liver and have been modified to produce, store and release insulin in response to glucose concentrations in the body. To manage T1D, the melligen cells are encapsulated in cellulose microcapsules by Cell-in-a-Box technology(50). According to the manufacturer, one of the most important features of microcapsules fabricated by Cell-in-a-Box technology is that they can be frozen and after thawing, cells with the viability of more than 95% could be attained (51). There is not a lot of information released about clinical trials on Cell-in-a-Box technology. However, evaluation of porcine islets encapsulated by Cell-in-a-Box technology in rats showed normal blood glucose levels for 6 months. In 2015, the press released, “This is the first time PharmaCyte Biotech will employ cells derived from humans (melligen cell line) in a preclinical study” and they were still in the preclinical stages until 2019 (51, 52). 

**Neurodegenerative disorders**

Neurodegenerative disorders like Parkinson’s disease (PD) constitute the other area that cell microencapsulation technology has entered. Using this technique, it is possible to overcome several obstacles in drug delivery to the CNS (*e.g*., BBB). LCT has invested in this area. They have encapsulated clusters of neonatal porcine choroid plexus cells in alginate microcapsules coated with PLO and called this product NTCELL^®^. Since NTCELL^®^ produces nervous growth factors, it could be helpful in neurodegenerative disorders. According to the result of phase I/II clinical trials, implantation of NTCELL^®^ was safe without any specific side effects (53). In the final cohort of phase IIb, which was conducted in New Zealand, 6 patients with PD participated (4 received NTCELL^®^ and 2 received no NTCELL^®^). None of the 6 patients showed any side effects. Phase IIb was conducted to confirm the most effective dose of NTCELL^®^. After 2 years of treatment, results of phase IIb showed that xenotransplantation was safe, and the results of the test group (the group with NTCELL^®^) were significantly different from the control group (the group with no NTCELL^®^). Stage III is the next level of clinical trials ahead of NTCELL^®^ (53, 54).

**Pancreatic cancer**

Pancreatic tumors respond to the chemotherapy limitedly, and they are often inoperable. PharmaCyte has used the Cell-in-a-Box technology to encapsulate genetically modified cells expressing cytochrome P450 and use them as a treatment in patients with pancreatic cancer. After implantation ofing these modified encapsulated cells in the body as close as possible to the tumor site, ifosfamide is given intravenously. It is designed that by reaching to the encapsulated cells, IV ifosfamide flows through pores in the capsules where it converts into the active form by the enzyme that is produced by genetically modified live cells. It is worth mentioning that using this strategy, the administered dose of ifosfamide could be cut to the one-third of the normal dose.

First, these modified cells were encapsulated in cellulose sulphate and evaluated in phase I/II clinical trials on 14 patients with pancreatic cancer. According to that study, in 4 patients, tumors regressed and in other participants who finished the study, no further tumor growth was recorded (55, 56). PharmaCyte has conducted phase I/II clinical trials to study the combination of Cell-in-a-Box technology with the prodrug (ifosfamide) administration on 27 patients with inoperable pancreatic cancer. The results showed that implantation was safe, and there was no immune response or inflammation towards encapsulated cells or materials. They conclude thatCell-in-a-Box combined with low-dose ifosfamide was safe and effective in patients with inoperable pancreatic cancer (57). Todate, PharmaCyte aims to initiate phase IIb clinical trial in locally advanced inoperable pancreatic cancer at trial sites throughout the US (58). 

Table 1 briefly shows the cell microencapsulated systems, which have reached industrial-scale production or clinical trials.

**Table 1:** Cell microencapsulated systems, which have reached industrial-scale production or clinical trials

**Cell microencapsulation technology /Product**	**Microcapsule-making material**	**Coating layer**	**Cell line**	**Current clinical trial stage**	**Ref**
ENCAPSEL^®^	Alg	PLL	Hybridoma cell line	-	(39, 40)
DIABECELL^®^	Alg	PLO	Islet cells from pathogen-free pigs	IIb	(42-44)
Cell-in-a-Box (T1D)	Cellulose	-	Melligen-human liver cells, genetically engineered to produce insulin	Preclinical	(50-52)
Cell-in-a-Box (Pancreatic cancer)	Cellulose	-	Genetically modified cells expressing cytochrome P450	IIb	(55-58)
NTCELL^®^	Alg	PLO	Neonatal porcine choroid plexus cells	III	(53, 54)
ENCAPTRA^®^	Polytetrafluoroethylene (PTFE)	-	VC-01-Beta-cell precursors	II	(47-49)

## Conclusion

Although due to the controversial results of different studies about cationic polymers, the literature review does not lead us to a consensus about the best cationic polymer as covering layer of alginate microcapsules. But by taking a closer look at the cell microencapsulated systems which are in industrial-scale production (ENCAPSEL^®^) or reached the human clinical trials (*e.g*., DIABECELL^®^), it may be concluded that, so far, PLL and PLO are the best and the most successful cationic polymers as covering layers of alginate microcapsules. As both of them are successful (PLL in ENCAPSEL^® ^and PLO in DIABECELL^®^ and NTCELL^®^), we may conclude that it is unnecessary to exclude PLL from this technique.

Another interesting point is that, contrary to the several articles in which alginate has been introduced as the best microcapsules’ making material, it can be seen that cellulose has also been used successfully to fabricate cell-loaded microcapsules; as discussed before, Cell-in-a-Box is a cellulose-based technology. These two polymers (alginate and cellulose) are in close competition.
